# TOR Complex 1: Orchestrating Nutrient Signaling and Cell Cycle Progression

**DOI:** 10.3390/ijms242115745

**Published:** 2023-10-30

**Authors:** Magdalena Foltman, Alberto Sanchez-Diaz

**Affiliations:** 1 Mechanisms and Regulation of Cell Division Research Unit, Instituto de Biomedicina y Biotecnología de Cantabria (IBBTEC), Universidad de Cantabria-CSIC, 39011 Santander, Spain; 2 Departamento de Biología Molecular, Facultad de Medicina, Universidad de Cantabria, 39011 Santander, Spain

**Keywords:** cell cycle, cell growth, TOR, TORC1, *S. cerevisiae*, budding yeast

## Abstract

The highly conserved TOR signaling pathway is crucial for coordinating cellular growth with the cell cycle machinery in eukaryotes. One of the two TOR complexes in budding yeast, TORC1, integrates environmental cues and promotes cell growth. While cells grow, they need to copy their chromosomes, segregate them in mitosis, divide all their components during cytokinesis, and finally physically separate mother and daughter cells to start a new cell cycle apart from each other. To maintain cell size homeostasis and chromosome stability, it is crucial that mechanisms that control growth are connected and coordinated with the cell cycle. Successive periods of high and low TORC1 activity would participate in the adequate cell cycle progression. Here, we review the known molecular mechanisms through which TORC1 regulates the cell cycle in the budding yeast *Saccharomyces cerevisiae* that have been extensively used as a model organism to understand the role of its mammalian ortholog, mTORC1.

## 1. Introduction

The cell cycle machinery needs to be coordinated with growth and metabolism to keep cell size homeostasis [1]. Over the last three decades, molecular details for such coordination have emerged, but our understanding is still limited. The highly conserved target of rapamycin (TOR) kinase plays a key role in the regulation of growth and proliferation in eukaryotes. TOR kinase forms two functionally distinct complexes, TORC1 (TOR complex 1) and TORC2 (TOR complex 2), of which only TORC1 is sensitive to rapamycin [2,3,4,5]. Mammalian cells present only a single form of the catalytic TOR kinase named mTOR (‘m’ stands for mechanistic or mammalian), which has serine/threonine protein kinase activity. Human mTORC1 is a dimer of mTOR-mLST8-RAPTOR heterotrimers [6,7,8]. In addition to mTOR kinase, the complex contains mLST8 (mammalian lethal with SEC13 protein 8 (also known as GβL) and the scaffold protein RAPTOR (regulatory-associated protein of mTOR), which plays a key role in the recognition of mTOR substrates and localization of mTORC1 [4,9,10,11,12]. mTORC1 contains two other factors, DEPTOR and PRAS40, for which there is no ortholog in yeast.

Instead of a single form, budding yeast cells have two kinases: Tor1 and Tor2 [2,13] (Table 1). Yeast TORC1 contains two other conserved factors: Kog1 (RAPTOR for mammals) and Lst8 (mLST8 for mammals) (Table 1). Kog1 recruits substrates to the Tor kinase and regulates its function, whereas Lst8 interacts directly with Tor1 and might stabilize the complex. In addition, budding yeast TORC1 contains a largely unknown factor named Tco89 for which there is no ortholog in mammals [2,3,13,14] (Table 1). TORC1 is primarily located on the vacuole membrane (vacuoles are the lysosomes in mammalian cells) [14,15,16]. However, Tor1 has also been identified in the nucleus [17]. On the other hand, TORC2 contains only the kinase Tor2 as the catalytic subunit in budding yeast. Lst8 is present in both TORC1 and TORC2. However, the rest of the yeast TORC2 components are distinct from those in TORC1: Avo1, Avo2, Avo3/Tsc11, Bit2 and Bit61 [5,18,19]. TORC2 has been described to be involved in the regulation of turgor pressure, plasma membrane tension, actin polarization, cell integrity pathway, G2/M cell cycle transition, genome integrity and pentose phosphate pathway [5,19,20].

**Figure 1:**
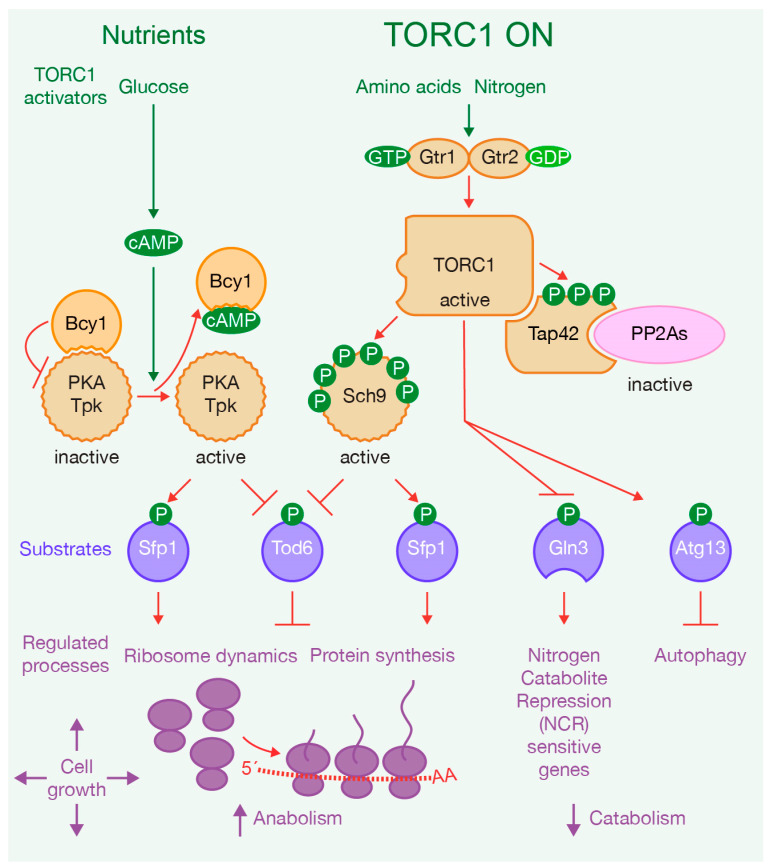
Nutrient availability drives cell growth via activation of TORC1 and PKA. The presence of nutrients triggers the activation of TORC1 and PKA. TORC1 phosphorylates two of its major effectors, kinase Sch9 and Tap42. Phosphorylated Tap42 interacts with phosphatases PP2A and PP2A-like (referred to in the illustration as PP2As), inhibiting their enzymatic activity, while all are bound to TORC1. The coordinated actions of TORC1, PKA, and Sch9 phosphorylate substrates inducing anabolism and suppressing catabolism.

**Figure 2:**
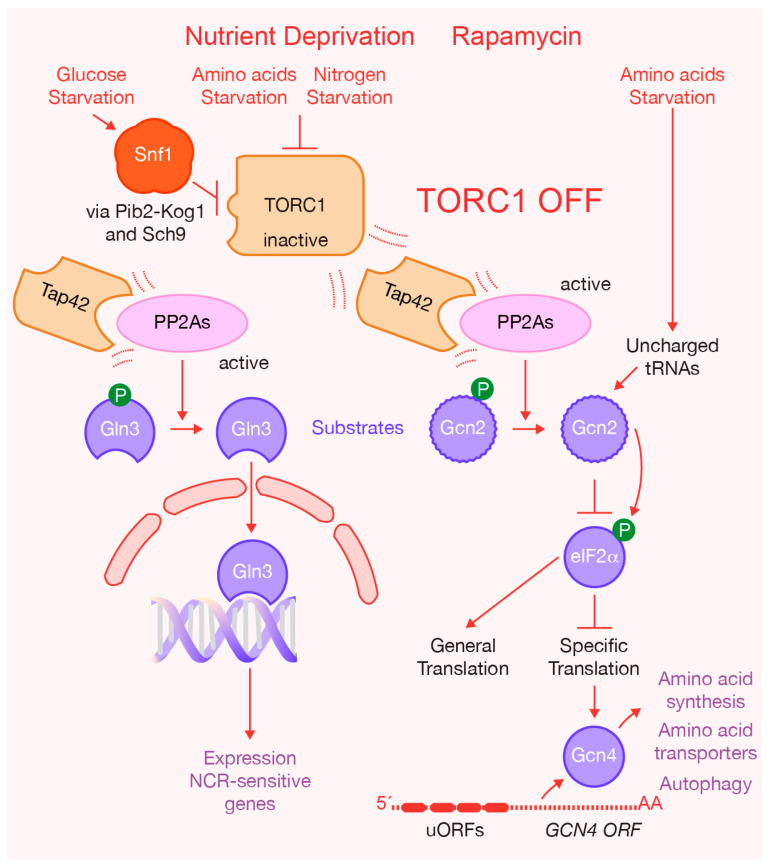
Inactivation of TORC1 promotes the activity of phosphatases PP2A and PP2-like (PP2As). Nutrient deprivation or addition of rapamycin blocks the kinase activity associated with TORC1, leading to Tap42 dephosphorylation. This weakens the interaction (shown by dotted lines around the key proteins), first between TORC1 and Tap42-PP2As complexes, and second between Tap42 and phosphatases responsible for dephosphorylating downstream factors such as Gln3 and Gcn2. Glucose starvation activates Snf1 kinase, inhibiting TORC1 activity.

In both yeast and mammals, TORC1 couples environmental signals, including nutrients availability, growth factors and hormones, to cellular growth by triggering anabolic processes such as ribosome dynamics, protein synthesis and metabolic pathways. These processes include ribosomal protein expression, ribosomal RNA synthesis, ribosome biogenesis, activation of dormant ribosomes, translation and transcription, as well as nucleotide and lipid synthesis. Simultaneously, TORC1 suppresses catabolic processes like autophagy [3,4,12,15,21,22,23,24,25,26,27,28]. Nutrients play a significant role as activators of TORC1 since they have the capacity to trigger TORC1 activation and, consequently, cell growth [3] (Figure 1). In yeast cells, the presence of glucose, amino acids and other nitrogen sources induces TORC1 activation, while the absence of these nutrients inhibits TORC1. Specifically, amino acids activate TORC1 through the small GTPases Gtr1 and Gtr2 (Figure 1), which are orthologs of the mammalian Rag GTPases. When amino acids are sufficient, Gtr1 is in an active conformation, loaded with GTP, while Gtr2 is loaded with GDP [3] (Figure 1). Glucose starvation, conversely, induces AMP-activated protein kinase (AMPK Snf1), which promotes TORC1 disassembly and the translocation of Kog1 to a single body, leading to a reduction in TORC1 functionality. The phosphorylation of Kog1 in a Snf1-dependent manner drives these changes [29,30] (Figure 2). Notably, Snf1 also regulates Pib2, a protein that binds to phosphatidylinositol-3-phosphate (PI3P) and to Kog1. Snf1 phosphorylates Pib2, weakening its association with Kog1 [31]. In coordination, Snf1 phosphorylates Sch9, a downstream effector of TORC1 (Figure 1 and Figure 2). This phosphorylation has an inhibitory effect on TORC1’s ability to phosphorylate and activate Sch9 [31]. Additionally, glucose activates cAMP-dependent protein kinase (PKA), which drives biological responses similar to TORC1 (Figure 1). The TORC1 pathway closely interacts with the PKA pathway to regulate ribosome dynamics and protein synthesis [32,33,34,35] (Figure 1). The PKA pathway is primarily activated by glucose and other fermentable sugars, and is required for most of the cells’ transcriptional responses to glucose [34]. When cells are exposed to glucose, they activate adenylate cyclase, leading to an increase in cAMP production. This cAMP binds to the regulatory subunit of PKA, Bcy1, which inhibits the activity of PKA’s catalytic subunit (Tpk1, 2, or 3). The binding of cAMP to Bcy1 promotes its dissociation from the catalytic subunit, thereby activating PKA [34] (Figure 1). Lastly, nitrogen sources activate TORC1 via glutamine synthesis, and consistently with this, blocking glutamine synthesis decreases TORC1 activity [36] (Figure 1). Nutrient deprivation or treatment with rapamycin inhibits TORC1 activity, ultimately inducing cellular quiescence [37].

TORC1 exerts its functions through downstream effectors, with two well-known examples in yeast being Sch9 and Tap42 (Figure 1). Sch9 is a member of the AGC kinase family and a functional ortholog of vertebrate S6 kinase (S6K). In the presence of nutrients, TORC1 phosphorylates Sch9, subsequently promoting ribosome dynamics and protein synthesis [15,38,39,40,41] (Figure 1). TORC1 also phosphorylates another downstream effector, Tap42 (Figure 1). Tap42 has the ability to interact with serine/threonine phosphatases PP2A and PP2A-like (referred to together in this review as PP2As). Upon phosphorylation by TORC1, Tap42 forms complexes with PP2As, which then associate with TORC1. This interaction leads to the inhibition of PP2As by TORC1, preventing these phosphatases from dephosphorylating TORC1 effectors [18,42,43,44]. The inactivation of TORC1 promotes Tap42 dephosphorylation, which weakens the association with phosphatases capable of dephosphorylating downstream factors. Therefore, TORC1 regulates the Tap42–phosphatase interaction in response to nutrient conditions [44,45] (Figure 2).

In addition to its major effectors, TORC1 has also been shown to phosphorylate other substrates directly. For instance, it phosphorylates Gln3, a transcription activator responsible for driving the expression of nitrogen catabolite repression (NCR) sensitive genes [46] (Figure 1). In nitrogen-rich conditions, Gln3 remains in the cytoplasm, preventing NCR-sensitive genes’ transcription (Figure 1). Conversely, in a nitrogen-limiting environment, Gln3 becomes activated as PP2As dephosphorylate it, causing Gln3 to relocate to the nucleus [47,48] (Figure 2). Recent research has found that the interaction between TORC1 and Gln3 is more intricate than previously understood [49]. Specifically, the C-terminal region of Gln3 is required for both its cytoplasmic retention and its rapamycin-dependent nuclear localization (Figure 2). Moreover, it has been determined that TORC1 interacts with the N-terminal region of Gln3, also playing a positive role in the regulation of Gln3 function [49].

Furthermore, TORC1 regulates the phosphorylation of the protein kinase Gcn2, which, in turn, controls translation initiation by phosphorylating and inhibiting the translation initiation factor eIF2 [50]. A lack of amino acids promotes the accumulation of uncharged tRNAs, which activates Gcn2 (Figure 2). Gcn2 activity is also modulated by phosphorylation. The inhibition of TORC1 function enhances the activity of Tap42–phosphatase-dependent dephosphorylation of Gcn2 (Figure 2). Subsequently, Gcn2 phosphorylates and inhibits the α subunit of the initiator factor eIF2, reducing the rate of general translation initiation, while inducing the specific translation of mRNA-encoding transcription factor Gcn4 [3,24,50,51] (Figure 2). Due to the presence of four short upstream open reading frames (uORFs) in its leader sequence, *GCN4* mRNA is selectively translated [3,24,50]. In conditions of amino acid starvation, Gcn4 promotes the expression of genes involved in the biosynthesis of amino acid precursors, amino acid transporters or autophagy proteins to enable the adaptation to the absence of amino acids [3,50] (Figure 2). Interestingly, as discussed further below, the role of uORFs in translation initiation differs between *GCN4* and *CLN3*, a key G1-progression regulator.

Additional substrates of the TORC1 pathway include transcriptional regulators of ribosomal proteins and ribosome biogenesis, such as Sfp1 activator and Tod6 repressor (Figure 1). Their nuclear-cytoplasm localization depends on their phosphorylation status [23,39,41,52]. Moreover, TORC1 activity suppresses autophagy via direct phosphorylation of Atg13 (Figure 1). Interestingly, a mutated version of Atg13 at these phosphorylation sites induces autophagy [53,54].

**Figure 3:**
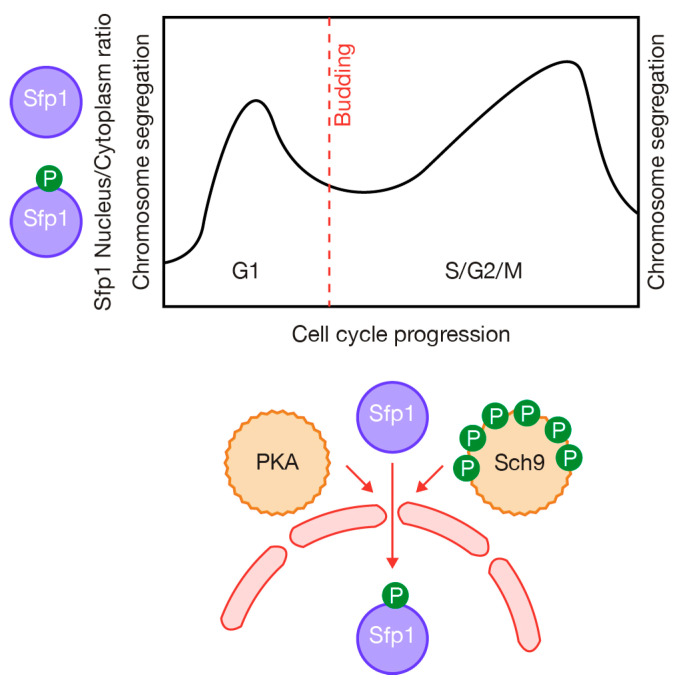
The kinase activity associated with TORC1 and PKA is regulated along the cell cycle. This illustration is adapted from the work of Guerra and colleagues, depicting the localization of the Sfp1 activator as an indicator of TORC1 and PKA function during the cell cycle [55]. TORC1 and PKA activities reach their peak during the G1 phase. Subsequently, there is a decrease in activity around the time of budding, followed by an increase during the S phase, G2 phase, and early mitosis. Activity is then downregulated in late mitosis. The study covered the period from chromosome segregation to the subsequent chromosome segregation [55].

Interestingly, by monitoring Sfp1 and Tod6 as reporters, Guerra and colleagues found that the activities of the TORC1 and PKA pathways fluctuate throughout the cell cycle, even under constant external condition [55] (Figure 3). TORC1 and PKA activities peak during the G1 phase, followed by a decrease around the time of budding. Subsequently, the activity increases during S, G2 and early mitosis followed by a decrease in late mitosis [55] (Figure 3). The importance of TORC1 during G1 was initially demonstrated as rapamycin inhibits translation initiation and protein synthesis, which promotes an arrest at the G1 phase of the cell cycle [56]. Moreover, the TORC1 and PKA cascades also converge at the Rim15 kinase to control entry into G0 [57], as detailed below. Similar findings to those of Guerra and coworkers were identified using single-cell experiments to investigate metabolic activity or amino acid synthesis [58,59].

TORC1 is a central coordinator of cell growth and the cell cycle machinery in all eukaryotic cells [60,61,62,63,64,65,66]. The kinase activity associated with Cyclin-Dependent Kinases (CDKs) drives cell cycle progression [67,68,69,70]. In budding yeast, Cdk1 (also known as Cdc28) serves as the main CDK. The catalytic subunit of the complex binds to various cyclins at different stages of the cell cycle and phosphorylates key substrates to perform chromosome replication, initiated by S-CDKs, or chromosome segregation, promoted by M-CDKs. Cyclins are expressed sequentially, contributing to the organization of the cell cycle and providing substrate specificity to ensure orderly cell cycle progression [71,72]. Furthermore, as cells progress from G1 to mitosis, the increasing kinase activity of CDKs prompts the transitions between different cell cycle phases as cells surpass specific thresholds of CDK activity [73,74,75]. Recent research has indicated that the substrate specificity for S-CDKs and M-CDKs is similar, with minor qualitative differences between the two CDK types [76]. Under certain conditions, an increase in S-CDK activity alone is enough to induce mitosis, effectively replacing the function of M-CDKs [76]. Consequently, cell cycle progression primarily results from a quantitative increase in kinase activity associated with CDKs, complemented by minor substrate specificity variations driven by cyclins [76].

The same kinase activity that promotes cell cycle is able to block late cell cycle events like exit from mitosis, cytokinesis and cell separation [77,78,79,80,81]. To complete each round of cell division, cells must downregulate CDKs. Furthermore, they must maintain a period of low CDK activity to assess environmental cues promoting cell growth, ensure proper replication origin licensing to prevent genomic instability, and create an opportunity for sexual differentiation [82,83,84].

Therefore, each cell cycle consists of alternating periods of high and low CDK activity. These phases are vital to ensure that each cell cycle phase occurs once and only once, maintaining the correct order of all phases [67,68,69,70]. Interestingly, as discussed earlier, TORC1 activity also fluctuates throughout the cell cycle [55,58,59]. Notably, TORC1 activity peaks in G1, earlier than CDK function. The oscillation between high and low TORC1 activity levels may play a crucial role in cell cycle regulation. In this review, we explore how TORC1 regulates the cell cycle in budding yeast.

## 2. TORC1 Promotes G1 Progression by Inducing G1 Cyclin Activity

Periodic transcription of genes, clustered according to their peak expression in G1, S, G2 or M phases, is crucial for successful progression through specific cell cycle phases and is evolutionarily conserved [85,86,87]. The expression of these gene clusters relies on the activity of specific Cyclin-CDKs that phosphorylate transcription factors regulating cell cycle-related transcription [87].

**Figure 4:**
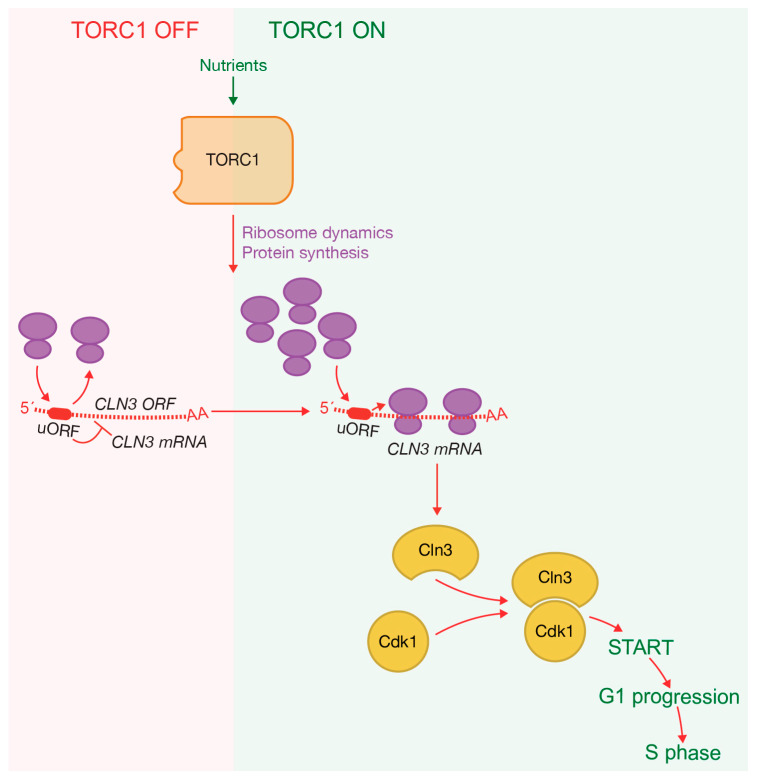
TORC1 promotes accumulation of Cln3-Cdk1 complexes, driving G1 progression. TORC1 regulates proteins involved in ribosome dynamics and protein synthesis, contributing to an increase in Cln3 protein levels that form complexes with Cdk1. Translation of *CLN3* is controlled by an upstream open reading frame (uORF), which inhibits efficient translation under nutrient-limited conditions when ribosome availability is reduced. The presence of rapamycin and nutrient starvation blocks kinase activity associated with TORC1.

In budding yeast, the G1 cyclin Cln3 drives the progression through the G1 phase of the cell cycle, enabling cells to reach a critical point known as START in yeast and Restriction Point in mammalian cells. This point marks a no-return stage, after which cells are committed to continuing their cell division cycle. During G1, cells grow and an increase in Cln3-Cdk complexes stimulates the expression of the G1/S cluster, facilitating entry into S-phase. This process depends on both cell growth and cell size [87,88,89]. Cln3 protein levels increase gradually throughout the G1 phase, peaking around the time of bud emergence, close to the onset of S-phase [90,91]. Passage through START is regulated by nutrient availability, ensuring that cells have the necessary resources to complete the cell cycle. The presence of fermentable carbon and nitrogen sources, which activate TORC1 function (Figure 1), is essential for achieving the highest induction of *CLN3* mRNA levels [92] (Figure 4). Notably, the inactivation of TORC1 leads to the downregulation of G1 cyclins and subsequently results in a G1 arrest [56,93].

The translation of *CLN3* is controlled by an uORF, encoding the tripeptide Met-Asp-Phe, which represses its translational efficiency if access to nutrients is limited [94] (Figure 4). Ribosomes can bypass the uORF through leaky scanning, leading to *CLN3* mRNA translation [94] (Figure 4). This limited *CLN3* mRNA translation is up-regulated when the general translation initiation factor eIF4E is over-expressed [95,96]. In *S. cerevisiae*, eIF4E was initially identified as a cell division cycle gene, *CDC33*, whose inactivation promotes a G1-arrest [97,98]. *CLN3* uORF promotes cell division in rich media and blocks it under slow growth. Inactivating the uORF enhances *CLN3* mRNA translational efficiency, shortening G1 and promoting rapid passage through START under slow growth, a phenotype also observed with *CLN3* overexpression [99]. The short half-life of Cln3 underscores the significance of translational control for regulating START completion.

The accumulation of sufficient Cln3 protein levels depends on optimal growth conditions. A shift from rich to poor conditions leads to a rapid decrease in Cln3 protein abundance [91]. TORC1-mediated stimulation of translation initiation promotes Cln3 accumulation, while rapamycin inhibits G1 progression by blocking translation initiation and reducing Cln3 synthesis [56] (Figure 4). Recent studies have shown that Cln3 levels are regulated by Ypk1 and Ypk2, the budding yeast homologs of human SGK kinases, which are components of the TORC2 signaling network. The inactivation of Ypk1/2 leads to a rapid and complete reduction in Cln3 levels, implying a significant role for TORC2 in the modulation of Cln3 levels [91]. Inactivating the uORF bypasses rapamycin-induced G1 arrest, highlighting its role in the sensing levels of protein synthesis [94]. This inhibitory mechanism is eluded when cells grow fast in optimal conditions. Interestingly, the inactivation of uORF is unable to avoid the cell cycle arrest induced when cells enter the stationary phase [94], suggesting the existence of additional mechanisms, beyond *CLN3* translation control, that coordinate environmental cues with the cell division machinery to block cell cycle progression, as explained below.

**Figure 5:**
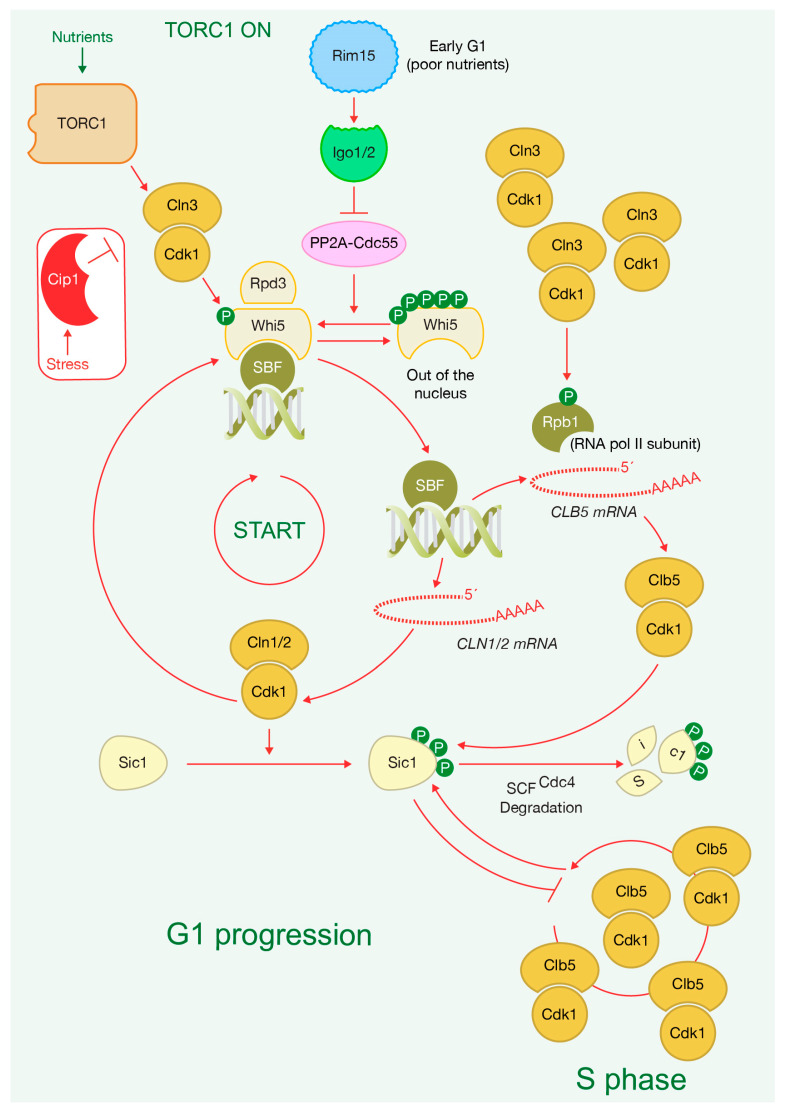
TORC1 drives G1 progression and ultimately entry into S phase. Transcriptional inhibitor Whi5 is recruited to G1/S promoters, when it is hypophosphorylated, to inhibit the transcription factor complex SBF. Hyperphosphorylation of Whi5 by Cln-Cdk1 complexes promotes dissociation of Whi5 with SBF, drives Whi5 out of the nucleus and SBF induces expression of G1/S genes, such as the G1 cyclins, *CLN1* and *CLN2*, and B-type cyclin *CLB5*. Activation of SBF starts a positive feedback loops that irreversibly commit cells to the cell cycle entry and passage through START. In addition, Cln3 activity removes the histone deacetylase Rpd3, which promotes cell cycle entry too. Furthermore, Cln3-Cdk1 directly phosphorylates and activates RNA Poll II subunit Rpb1 to initiate transcription of G1/S genes. Accumulation of CDK inhibitor Cip1 prevents Whi5 phosphorylation by targeting mainly Cln3-CDK1 complexes, which induces early G1 arrest environmental stress. Sic1 dynamics controls G1 progression and entry into chromosome replication. Sic1 accumulates at the end of mitosis and is present throughout the G1 phase of the cell cycle. Cln-Cdk1 complexes escape from Sic1 inhibition and phosphorylate Sic1, which promotes the degradation of Sic1 via the SCF E3 ubiquitin-ligase. In addition, Clb5-Cdk1 complexes phosphorylate Sic1 and target Sic1 for degradation, which generates a positive feedback that contributes to Sic1 degradation and promotes G1-S.

The transition from G1 to S phase requires the inactivation of a transcriptional inhibitor that enables the expression of genes under the control of the SBF transcription factor complex, comprising DNA-binding protein Swi4 and modulator Swi6 [88]. In budding yeast, this inhibitor role is played by Whi5, which is recruited to G1/S promoters, where it interacts and blocks SBF during early G1 [100,101] (Figure 5). Phosphorylation by CDK complexes associated with Cln-type cyclins disrupts the Whi5-SBF association, and drives Whi5 out of the nucleus, promoting the induction of G1/S genes [100,101,102] (Figure 5). The precise timing of START is determined by Whi5’s nuclear export [103]. Activation of SBF transcription complex initiates positive feedback loops that irreversibly commit cells to the cell cycle entry. Downstream CDK activity phosphorylates Whi5, further reinforcing cell cycle progression [88] (Figure 5). The accumulation of the CDK inhibitor Cip1 mainly targets Cln3-Cdk1 complexes, preventing Whi5 phosphorylation and leading to an early G1 arrest in response to environmental stress [104] (Figure 5). Two other G1 cyclins, Cln1 and Cln2, are among the genes induced by Cln3 and contribute to the positive feedback loop [105,106] (Figure 5). Therefore, G1 cyclins induce their own transcription to promote a rapid increase in CDK activity (Figure 5). It has been recently proposed that Cln3-Cdk1 may not contribute to Whi5 hyperphosphorylation in early G1, with Cln1-Cln2-Cdk1 playing a more prominent role in this process [101,107,108] (Figure 5). Instead, Cln3-Cdk1 complexes directly phosphorylate and activate RNA Poll II subunit Rpb1 to initiate G1/S associated transcription [108] (Figure 5). Therefore, the levels of Cln3, controlled by TORC1, regulate G1 progression and directly influence the expression of specific genes. Additionally, the recruitment of Cln3 to promoters displaces the previously bound histone deacetylase Rpd3, promoting cell cycle entry by inducing the expression of S-phase genes [109] (Figure 5). Furthermore, it has been proposed that Whi5 is diluted as cells grow, unlike other G1/S regulators that seem to maintain a constant concentration as cells grow in G1, which might trigger START [110]. The increase in the expression of most RNAs is proportional to cell growth, but there are key exceptions that regulate cell cycle progression and determine cell size at the G1/S transition. The concentration of key mRNAs for cell cycle activators, like Cln3, increases as cells grow larger, whereas other mRNAs for cell cycle inhibitors, like Whi5, decrease as cells grow. The balance between them determines cell size at the onset of chromosome replication [111]. However, other models proposed that an increase in global protein synthesis in late G1 promotes passage through START via the accumulation of Cln3 and phosphorylation inactivation of Whi5 [112,113,114]. Both models remain contentious [114,115]. The molecular mechanism that controls START seems to be more complex than just the level of two proteins. 

During the vegetative cell cycle, the phosphatase PP2A, along with its activator Cdc55, promotes the dephosphorylation of Whi5, as demonstrated by Talarek and coworkers. [116]. This action prevents cells from progressing through START. The Rim15-Igo1,2 pathway, which blocks the activity of PP2A^Cdc55^, as described below, becomes active when cells are grown under nutrient-poor conditions, facilitating passage through START (Figure 5). In early G1, the kinase activity associated with Cln3-Cdk1 complexes is low, resulting in limited Whi5 phosphorylation. Rim15-Igo1,2 prevents the dephosphorylation of Whi5, which in turn stimulates SBF-driven transcription. This shift in balance leads to increased phosphorylation of Whi5 by Cln3-Cdk1 [116] (Figure 5).

## 3. TORC1 Activity Ultimately Determines Sic1 Degradation to Drive Cells into S Phase

TORC1 couples cell growth with early cell cycle progression by regulating the protein stability of the CDK inhibitor Sic1, which blocks CDK complexes containing B-type cyclins, preventing entry into S-phase [117,118] (Figure 5). Sic1 accumulates in late M phase, persists through G1, and undergoes degradation to enable the progression into S-phase [118,119] (Figure 5). Sic1 contributes to CDK downregulation, enabling cells’ exit from mitosis, cytokinesis and cell separation [80,81,119,120]. Additionally, Sic1 plays an key role in the regulation of DNA replication as Sic1 needs to be degraded for Clb-CDK complexes to initiate S-phase entry [118] (Figure 5). Notably, Sic1 prevents untimely activation of CDKs, ensuring a period of low Clb-CDK activity. This allows for a proper replication origin licensing and subsequently adequate chromosome segregation, preventing genomic instability [121]. The deletion of *CLB5* and *CLB6* rescues defects associated with the lack of *SIC1*, which shows the important relationship between them [121]. Therefore, cells need to inactivate Sic1 to enter chromosome replication (Figure 5).

TORC1 induces the activity of Cln-Cdk1 complexes that escape from Sic1 inhibition but can promote the phosphorylation and degradation of Sic1 (Figure 5). Interestingly, a lack of Sic1 rescues defects associated with the triple deletion of G1 cyclins, showing their interdependence [122]. Cln-Cdk complexes initiate phosphorylation of Sic1 at its N-terminus, which generates specific docking sites for the phosphoacceptor factor Cks1 of Cln-Cdk, and for incipient Clb5/6-Cdk complexes [123]. Subsequently, Sic1 is phosphorylated in multiple sites to promote its destruction (Figure 5). Clb5-Cdk creates a positive feedback to ensure Sic1 degradation [123,124] (Figure 5). Cyclins provide substrate specificity to Cyclin-CDK complexes as several cyclins recognize docking motifs that induce specific substrate phosphorylation [125,126]. Early in the cell cycle, cyclins Cln1 and Cln2, but not Cln3 or Clbs, preferentially interact with specific docking motifs named LP motifs enriched in leucine and proline. Sic1 phosphorylation by Cln2-Cdk1 depends on the presence of LP motif [125], ensuring phosphorylation for cell cycle progression. Once phosphorylated, Sic1 is recognized and binds to SCF (Skp1-Cul1-F-box)-Cdc4 E3 ubiquitin-ligase, which promotes Sic1 ubiquitylation and degradation by the proteasome [123,127,128,129,130,131] (Figure 5). Consequently, kinase activity associated with Clb-Cdk complexes increases, promoting S-phase entry [124] (Figure 5).

## 4. Inactivation of TORC1 Promotes Sic1 Stabilization to Arrest the Cell Cycle

**Figure 6:**
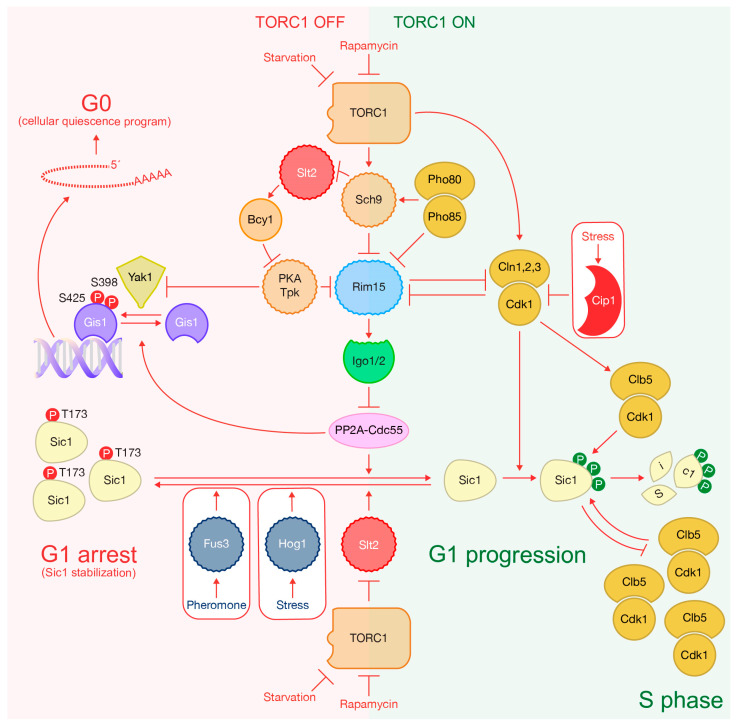
Inactivation of TORC1 promotes Sic1 stabilization to arrest the cell cycle. TORC1 coordinates cell cycle progression and nutrient availability in G1 through Sic1 phosphorylation on T173. This phosphorylation leads to the accumulation of Sic1 as it prevents its degradation, resulting in G1 arrest. Inactivation of TORC1 after the addition of rapamycin promotes a decrease in cyclin levels. Initially, a reduction in Cln1, Cln 2 and Cln 3, and consequently Clb5, reduces Sic1 phosphorylation and degradation. Clb5-Cdk1 complexes. Stress or pheromone-activated kinases, namely Slt2, Hog1 and Fus3, induce phosphorylation on T173, leading to a cell cycle arrest. Conversely, TORC1 drives a conserved signaling pathway that controls the activity of Cdc55 protein phosphatase 2A (PP2A^Cdc55^) to dephosphorylate pT173 within Sic1, promoting G1 progression. This signaling cascade includes kinase Rim15, which phosphorylates endosulfines (Igo1/2) to inhibit the PP2A^Cdc55^ directly. Both Rim15 and endosulfines are activated after nutrient limitation. Rim15 function is negatively regulated by TORC1, PKA, Pho80/Pho85 and Cln1,2,3-Cdk1. Additionally, PP2A^Cdc55^ dephosphorylates the transcriptional activator Gis1, preventing a gene expression program to drive cells into quiescence. The kinase Yak1 positively regulates Gis1 by inducing its phosphorylating and subsequently translocation to promoters. Furthermore, PKA counteracts the quiescence program by phosphorylating and inhibiting Yak1. The balance between Yak1 and PP2A^Cdc55^ regulates Gis1 to promote a reversible cellular quiescence program.

TORC1 inactivation leads to the phosphorylation of Sic1 at threonine residue T173 located in its C-terminus, resulting in Sic1 stabilization [93,132,133,134] (Figure 6). Sic1 stabilization can also be triggered by two other signaling pathways in response to stress or mating pheromone presence. The stress-activated protein kinase Hog1 and the pheromone-pathway MAPK Fus3 phosphorylate Sic1 at T173 [132,135] (Figure 6). This phosphorylation promotes the accumulation of Sic1, preventing its degradation by interfering with the interaction between Sic1 and the SCF E3 ubiquitin-ligase [132] (Figure 6). Rapamycin-induced TORC1 inactivation reduces G1 cyclin levels, reducing Sic1 phosphorylation and degradation (Figure 6). Furthermore, rapamycin increases Sic1 protein levels and its accumulation in the nucleus, which mediates G1 arrest [93]. This arrest requires Sic1 phosphorylation on residue T173 [93] (Figure 6). A version of Sic1 with a T173A mutation destabilizes Sic1 and compromises the G1 arrest in the presence of rapamycin [93]. 

The mitogen-activated protein kinase Slt2/Mpk1 regulates and participates in the phosphorylation of Sic1 on T173 [133] (Figure 6). Slt2 is the most downstream kinase of the so-called cell wall integrity (CWI) pathway that is activated after a variety of stresses including nutrient limitation or rapamycin treatment [136]. In fact, rapamycin increases the activity of Slt2 towards residue T173 and promotes the interaction between Slt2 and Sic1 [133]. Rapamycin also induces Slt2 activity as TORC1 indirectly inhibits Slt2 [137].

Phosphorylation of Sic1 at T173 creates a docking site for CDK phosphoacceptor factor Cks1. Together with a Clb5-binding RXL motif located at the C-terminus of Sic1, this interaction sequesters Clb5-Cdk complexes and prevents Sic1’s N-terminal phosphorylation. This dual mechanism prevents Sic1’s ubiquitin-dependent degradation and converts it into an inhibitor for Clb5-Cdk [134] (Figure 6). 

A reduction in TORC1 signaling, induced by carbon or nitrogen limitations, leads to G1 arrest and entry into quiescence [37,138] (Figure 6). This arrest is prompted by the decrease in CDK activity and the accumulation of Sic1 phosphorylated at the C-terminus as described above (Figure 6). On the other hand, the inactivation of TORC1 blocks the function of phosphatase PP2A^Cdc55^, preventing the dephosphorylation of pT173 within Sic1, which precludes its own degradation via the proteasome (Figure 6). As a result, Sic1 is stabilized, which leads to a cell cycle arrest at the G1 phase [133] (Figure 6). Therefore, TORC1 clearly coordinates G1 progression with nutrient availability. That TORC1-regulated pathway is the analogous to the greatwall kinase in *Xenopus*, the yeast protein kinase Rim15 that phosphorylates endosulfines (Igo1/2) to directly inhibit the PP2A^Cdc55^ [139,140] (Figure 6). Functional depletion of TORC1 by rapamycin addition induces rapid changes in the Rim15 phosphorylation profile, and, subsequently, the nuclear accumulation of Rim15 and Rim15-dependent transcription. TORC1 indirectly elicits Rim15 phosphorylation to promote its interaction with 14-3-3 proteins Bmh1/2, which blocks Rim15 nuclear entry. Simultaneously, TORC1 effector protein kinase Sch9 directly phosphorylates Rim15 to promote nuclear exclusion of Rim15 [57,141] (Figure 6). Additionally, nutrient limitation downregulates PKA, which releases Rim15 inhibition and drives cells into G0 in a similar way as occurs after the addition of rapamycin [142] (Figure 6). TORC1 signaling also positively regulates PKA via Sch9, which phosphorylates and inhibits Slt2. Subsequently, Slt2 is unable to phosphorylate and activate Bcy1, the negative regulatory subunit of PKA, which limits the activity of Bcy1 [21,143] (Figure 1 and Figure 6). Additionally, phosphate starvation drives entry into G0, which is controlled by the kinase activity associated with complexes formed by cyclin Pho80 and CDK Pho85. These complexes directly phosphorylate Sch9, preparing this kinase for subsequent phosphorylation by TORC1 [144] (Figure 6). Furthermore, Pho80/Pho85 complexes also phosphorylate Rim15 on the same residue as TORC1, promoting the accumulation of Rim15 in the cytoplasm where it is unable to activate gene expression to determine entry into quiescence [145,146] (Figure 6). Therefore, Rim15 is the key factor that integrates signals from TORC1, PKA and Pho80/Pho85 cascades, which explains how nutrient availability controls cell cycle progression (Figure 6). Interestingly, Cln1,2,3-Cdk1 negatively regulate Rim15 in cooperation with TORC1 by stimulating Rim15 localization in the cytoplasm, which contributes to the passage through START in proliferating cells [116,134] (Figure 6).

In addition to Sic1 dephosphorylation of pT173 [133], PP2A^Cdc55^ dephosphorylates the transcriptional activator Gis1, which inhibits its recruitment to promoter regions that drives cells into quiescence [139,147] (Figure 6). Furthermore, kinase Yak1 directly phosphorylates Gis1 to translocate it to those promoters [140]. Furthermore, PKA phosphorylates Yak1 to inhibit the nuclear localization of Yak1 and, therefore, its function [148]. Yak1 and PP2A^Cdc55^ form a kinase/phosphate couple that regulates Gis1 to promote a reversible cellular quiescence program in yeast (Figure 6).

## 5. TORC1 Regulates Mitosis Progression

**Figure 7:**
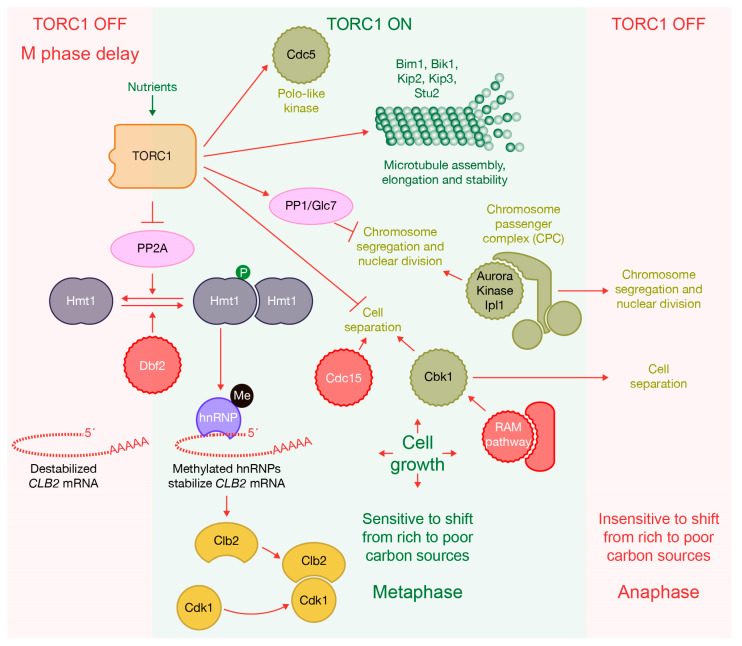
TORC1 controls mitosis progression and cell separation. B-type cyclin Clb2 is required to promote mitosis. TORC1 drives accumulation of *CLB2* transcript by inhibiting PP2A phosphatase Pph22, which dephosphorylates the arginine methyltransferase Hmt1. On the other hand, Dbf2 kinase counteracts PP2A phosphatase Pph22 function by phosphorylating Hmt1, which induces its oligomerization and activation. Hmt1 methylates heterogeneous nuclear RNA-binding protein (hnRNP), which stabilizes *CLB2* transcripts and promotes nuclear export. Inactivation of TORC1 after starvation or addition of rapamycin promotes PP2A phosphatase Pph22 to dephosphorylate Hmt1, which becomes inactive. Subsequently, Hmt1 is unable to methylate hnRNPs, preventing the accumulation of *CLB2* mRNA in mitosis. TORC1 positively controls other aspects of mitosis, like the nuclear localization of polo-like kinase Cdc5, and microtubule assembly, elongation and stability. Moreover, TORC1 activity seems to block chromosome segregation and nuclear division by altering the function of mitotic Aurora kinase Ipl1, a component of the chromosome passenger complex (CPC). Interestingly, TORC1 regulates and participates in the phosphorylation of Cbk1, the most downstream factor of the RAM signaling cascade, to block cell separation before cells exit from mitosis, which contributes to the ordering of the different steps during the cell cycle and to the coordination of environmental cues with the cell cycle machinery. In addition, mitotic kinase Cdc15, a component of the MEN signaling cascade, counteracts TORC1 function by promoting cell separation. Metaphase cells are sensitive to a change from rich to poor carbon sources, inducing a metaphase delay, while anaphase cells appear to be unaltered by the shift as they are unable to delay mitosis.

Cells need to increase the kinase activity associated with CDKs to allow cells to segregate their chromosomes [67,68,69,70]. Mitotic cyclins are sequentially expressed, which contributes to the driving of different mitotic events [71,72]. The transcription of one of those mitotic cyclins, *CLB2*, is controlled and increases as cells become closer to mitosis. Subsequently, entry into mitosis promotes rapid *CLB2* mRNA decay that, if prevented, would avoid exit from mitosis [149,150]. The half-life of *CLB2* drops more than 30-fold before metaphase. A change in *CLB2* mRNA stability is driven by mitotic exit network kinases Dbf2 and Dbf20 [151], which, in addition, are involved in the depletion of Clb2 protein to downregulate kinase activity associated with mitotic Cyclin-CDK complexes. Interestingly, Dbf2 has also been described to promote an increase in *CLB2* transcript to drive mitosis [152] (Figure 7). Therefore, it seems that Dbf2 has a dual role in promoting the stability and the decay of *CLB2* mRNA. Dbf2 phosphorylates the arginine methyltransferase Hmt1, which induces its oligomerization and activation (Figure 7). In turn, Hmt1 methylates heterogeneous nuclear RNA-binding proteins (hnRNP), which stabilize *CLB2* transcripts and promote nuclear export. 

To counteract Hmt1 activity and induce the decay of *CLB2* mRNA, the catalytic subunit of the PP2A phosphatase, Pph22, promotes Hmt1 dephosphorylation [152]. Consequently, the balance between the functions of Dbf2 and Pph22 regulates the entry into mitosis (Figure 7). The timing of chromosome segregation is delayed following the addition of rapamycin or the inactivation of components of TORC1 in budding yeast [153], which shows again a connection between the machinery that promotes cell cycle progression and the environmental conditions (Figure 7). When TORC1 is inhibited by rapamycin or during starvation, it triggers the recruitment of Pph22 to Hmt1. Subsequently, Hmt1 undergoes dephosphorylation, leading to the dissociation of Dbf2 from Hmt1. As a result, Hmt1 becomes inactive, preventing the methylation of hnRNPs and ultimately avoiding the accumulation of *CLB2* mRNA in mitosis [152] (Figure 7).

Environmental signals promote the extension of critical cell cycle transitions to maintain cell size [154]. In fact, a change from rich to poor carbon sources induces metaphase delay. In contrast, anaphase cells appear to be unaffected by the shift as they are unable to delay mitosis [155] (Figure 7). This observation is consistent with recent findings that the kinase activity associated with TORC1 and PKA is downregulated in late mitosis [55,58,59]. We have very recently shown that TORC1 is inactivated in anaphase before Clb2 inactivation is started at the anaphase to telophase transition [156]. Furthermore, the addition of rapamycin to inactivate TORC1 in G2-M arrested cells is enough to promote the dephosphorylation of the TORC1 substrate Gln3. This would indicate that Gln3 dephosphorylation depends on TORC1 downregulation and occurs earlier than Clb2 depletion [156]. Therefore, anaphase cells could potentially disable the mechanism responsible for coordinating the progression of the cell cycle and cellular growth. It has been described that the inactivation of TORC1 increases mitotic slippage after prolonged metaphase block [157], although we have shown that the downregulation of TORC1 in anaphase-arrested cells is unable to promote mitotic exit [156]. Interestingly, TORC1 is inactivated during mitosis in human cells too [158], which might indicate that TORC1 activity during the cell cycle is regulated in a similar way in eukaryotes.

This tight control over the kinase activity associated with TORC1 is reminiscent of how CDK activity fluctuates throughout the cell cycle. CDK is able to phosphorylate key substrates to promote cell cycle progression and, at the same time, phosphorylate other substrates to block their function late in the cell cycle. This assures the orderly sequence of events and that they take place only once per cell cycle [67,69,70]. Both activities, TORC1 and CDK, need to be downregulated to enable the last steps in the cell cycle (Figure 7).

Moreover, TORC1 regulates other aspects of mitosis in budding yeast like the nuclear localization of one of the main mitotic kinases, the polo-like kinase Cdc5, which is essential for its function (Figure 7). The overexpression of Cdc5 rescues the growth defect associated with a TORC1-defective mutant [153]. The PP2A phosphatase contributes to the localization and function of Cdc5 during mitosis [153]. Furthermore, the inactivation of TORC1 using rapamycin promotes problems in microtubule assembly, elongation and stability (Figure 7). In addition, rapamycin induces defects on spindle orientation, nuclear movement and positioning, karyogamy and chromosomal stability [159,160]. This function seems to be coordinately shared with cyclin Clb5 through kinesin-like motor protein Kip3 [160]. TORC1 interacts with Bik1, a highly conserved plus end-tracking protein (+TIPs) that specifically recognizes growing microtubule plus ends and plays a key role in microtubule organization [159]. It has been recently shown that Bik1 stabilizes Kip2, a kinesin that promotes microtubule growth, which might be one of the ultimate TORC1 functions in microtubule dynamics to promote mitotic early steps [161]. Another conserved +TIP protein, Bim1, interacts with TORC1 [162]. In addition, TORC1 drives phosphorylation of the microtubule polymerase Stu2, which prevents the nuclear accumulation of Stu2 before mitosis and participates in cytoskeletal organization in interphase and mitosis. The interaction between TORC1, Bim1 and Bik1 facilitates TORC1-dependent phosphorylation of Stu2 [162]. Another connection between TORC1 and mitotic regulatory machinery is through the essential mitotic Aurora kinase Ipl1, which associates with three other proteins to form the chromosome passenger complex (CPC) [163] (Figure 7). Decreased TORC1 activity rescues defects in chromosome segregation in an *ipl1* mutant, most likely by modulating the function of the PP1 phosphatase, Glc7, which counteracts Ipl1-mediated substrate phosphorylation during mitosis. Interestingly, a lack of TORC1 component Tco89 reduces nuclear accumulation of the Glc7 [164]. While it is clear that TORC1 regulates different aspects of mitosis, molecular details are still poorly understood.

## 6. TORC1 Blocks Separation between Mother and Daughter Cells

The elongation of anaphase spindle allows for chromosome separation and triggers the activation of the budding yeast mitotic exit network (MEN), which promotes mitotic exit, cytokinesis, cell separation and entry into a new G1 phase [79,165]. During cytokinesis, actomyosin ring contraction and plasma membrane ingression are coordinated with the formation of extracellular matrix, which is referred to as the septum in yeast [166,167,168]. To allow for the separation between mother and daughter cells, it is essential that part of that septum is digested. This process is driven by the daughter cell that is able to specifically secrete hydrolytic enzymes, allowing the two cells to separate [80]. In budding yeast, the RAM (Regulation of Ace2 and Morphogenesis) signaling pathway promotes cell separation at the end of the cell cycle [80] (Figure 7). MEN and RAM cascades share organizational and structural components and both are functionally distinct conserved “Hippo” signaling, which regulates cell growth, proliferation and morphogenesis in eukaryotes [80]. The NDR/LATS kinase Cbk1 is the central regulatory component of the RAM network and phosphorylates the transcription factor Ace2 in anaphase, which drives the expression of hydrolytic enzymes that are secreted at the site of division to promote cell separation [169,170,171]. We have very recently found that TORC1 regulates and participates in the phosphorylation of Cbk1 to block cell separation before cells exit from mitosis, which contributes to order the different steps during the cell cycle and to coordinate environmental cues with the cell cycle machinery [156] (Figure 7). Interestingly, MEN kinase Cdc15 counteracts TORC1-dependent negative regulation of cell separation (Figure 7). In addition, we have described how TORC1 ultimately regulates the fusion of secretory vesicles transporting hydrolases at the site of division by controlling the kinase activity associated with Cbk1 [156]. Cbk1 binds to and phosphorylates the exocyst component Sec3, which is able to activate the SNARE complex to promote membrane fusion. TORC1-driven inactivation of Cbk1 alters the dynamics between Sec3 and a t-SNARE component, which prevents vesicle fusion and induces a cell separation defect [156]. Finally, cells would need to downregulate TORC1 activity in metaphase to allow for cell separation (Figure 7). In this way, cells order different steps during cell cycle progression. TORC1 would also guarantee the maintenance of a consistent size in the cell population from generation to generation before mother and daughter cells separate and they start a new cell cycle.

## 7. Concluding Remarks

TORC1 is involved in regulating various stages of the cell cycle across eukaryotes, which suggests that the importance of TORC1 control has been conserved throughout evolution to coordinate cell growth with cell cycle. Further investigation is required to gain a deeper comprehension of this coordination, especially to determine how that coordination occurs during the S-phase and cytokinesis. From other yeast to mammalian cells, TORC1 governs different steps of the cell cycle, including mitosis [62,66,172]. Kinase activity associated with CDKs fluctuates along the cell cycle to control the orderly sequence of phases and that they only occur once per cell cycle. In the same way, TORC1’s kinase activity varies during the cell cycle, which might contribute to cell cycle progression and the maintenance of the correct phase order. For instance, in budding yeast, TORC1 seems to be able to promote early mitosis and to block chromosome segregation or cell separation [152,156]. It seems that mTORC1 activity in mammalian cells might be regulated in a similar way to that in yeast since mTORC1 is also downregulated in mitosis [158].

Intriguingly, TORC1 function is tightly coordinated with other signaling pathways such as the Hippo pathway, which regulates cell proliferation, apoptosis and differentiation [64,173,174,175,176,177,178]. The deregulation of human mTORC1 is linked to pathologies such as cancer, obesity, type 2 diabetes and neurodegeneration, which shows the importance of TORC1 in cell biology [12,26,179,180]. Much has been learned over the last three decades. Studying budding yeast should continue to drive our understanding of how TORC1 regulates the eukaryotic cell cycle, which will be crucial for comprehending human diseases and potentially lead to innovative approaches in addressing disorders linked to the mTOR pathway.

## Figures and Tables

**Table 1:** Components of the TOR complex 1 (TORC1) in budding yeast.

*S. cerevisiae*	Function
Tor1 or Tor2	Serine/threonine protein kinases
Kog1	Recruitment of substrates to the Tor kinase and regulation of Tor kinase function
Lst8	Stabilize the complex
Tco89	
